# Methods to prevent PCR amplification of DNA from non-viable virus were not successful for infectious laryngotracheitis virus

**DOI:** 10.1371/journal.pone.0232571

**Published:** 2020-05-22

**Authors:** Yugal Raj Bindari, Stephen W. Walkden-Brown, Priscilla F. Gerber

**Affiliations:** Animal Science, School of Environmental and Rural Science, University of New England, Armidale, NSW, Australia; Panstwowy Instytut Weterynaryjny - Panstwowy Instytut Badawczy w Pulawach, POLAND

## Abstract

Molecular-based testing of poultry dust has been used as a fast, sensitive and specific way to monitor viruses in chicken flocks but it provides no information on viral viability. Differentiation of viable and nonviable virus would expand the usefulness of PCR-based detection. This study tested three treatments (1. DNAse, 2. propidium monoazide [PMA], 3. immunomagnetic separation [IMS]) applied to dust or virus stock prior to nucleic acid extraction for their ability to exclude nonviable virus from PCR amplification. Infectious laryngotracheitis virus (ILTV) was used as a model. These treatments assume loss of viral viability due to damage to the capsid or to denaturation of epitope proteins. DNAse and PMA assess the integrity of the capsid to penetration by enzyme or intercalating dye, while IMS assesses the integrity of epitope proteins. Treatments were evaluated for their ability to reduce PCR signal, measured as ILTV log_10_ genomic copies (ILTV GC), of heat and chemically inactivated ILTV in poultry dust and virus stock. Compared to untreated dust samples, there was an overall reduction of 1.7 ILTV GC after IMS treatment (p<0.01), and a reduction of 2.0 ILTV GC after PMA treatment (p<0.0001). DNAse treatment did not reduce ILTV GC in dust (p = 0.68). Compared to untreated virus stocks, there was an overall reduction of 0.5 ILTV GC after DNAse treatment (p = 0.04), a reduction of 1.8 ILTV GC after IMS treatment (p<0.001) and a reduction of 1.4 ILTV GC after PMA treatment (p<0.0001). None of the treatments completely suppressed the detection of inactivated ILTV GC. In conclusion, treatments that use capsid integrity or protein epitope denaturation as markers to assess ILTV infectivity are unsuitable to accurately estimate proportions of viable virus in poultry dust and virus stocks.

## 1. Introduction

A promising trend in the monitoring of incursion of pathogenic viruses and administration success of live virus vaccines in large poultry flocks is the use of PCR-based assays using poultry dust as a diagnostic sample [1–3]. This population-level sampling approach consists of collection of dust samples accumulated in settle plates or scraped from poultry shed fixtures for PCR testing [1, 3–5]. Although this method offers advantages over individual bird sampling such as ease of sample collection, a limitation is the inability to differentiate between viable and nonviable viruses by PCR due to the persistence of DNA in fully or partially intact viruses [6, 7]. This discrimination is important to assess epidemiological risks associated with virus transmission when using environmental samples for disease monitoring.

To overcome this issue, several viability-PCR approaches have been developed to selectively detect viable viruses from environmental samples [8, 9]. These methods are based on the assumption that virus inactivation is associated with loss of integrity of the viral outer structures (e.g. envelope and capsid), or with loss of the integrity of epitope proteins necessary for virus entry in the host cell. Loss of integrity of the viral outer structures can be evaluated using an enzyme (e.g. DNAse) or an intercalating dye (e.g. propidium monoazide [PMA]) treatment prior to the nucleic acid extraction to gain access to the nucleic acids within. In capsid compromised viruses, DNAse penetrates the virus particles and digests unprotected DNA [6, 10], while PMA binds DNA irreversibly upon photoactivation. Both methods prevent DNA from amplifying during the PCR assay [11]. Virus inactivation may also occur due to loss of the integrity of epitope proteins without the loss of capsid integrity, which can be assessed by immunomagnetic separation (IMS). In this case, antibodies specific to viral antigens are used to bind viruses with intact epitopes, while unbound viruses are washed out prior to extraction and thus only viable viruses are detected by PCR. IMS combined with PCR is commonly used to detect enteric viruses from environmental water samples [12–14].

In this study, DNAse, PMA, and IMS treatments applied prior to nucleic acid extraction were evaluated in their ability to prevent PCR amplification of inactivated *Gallid alphaherpesvirus 1*, also known as infectious laryngotrachetitis virus (ILTV), in poultry dust and virus stocks. This virus belongs to the subfamily *Alphaherpesvirinae*, family *Herpesviridae* and genus *Itovirus* [15] and causes an acute upper respiratory disease of chickens that leads to significant losses to the poultry industry [16]. ILTV contains a double stranded DNA genomic material encased in an icosahedral capsid surrounded by a large amount of globular proteins (tegument) and an outer envelope which contains glycoproteins [16].

Several molecular methods have been described to detect ILTV DNA in clinical samples [17], and in poultry dust [2] but those methods cannot differentiate between viable and non-viable ILTV. In an attempt to overcome this limitation, we optimised and assessed DNAse, PMA and IMS treatments on their ability to extinguish ILTV PCR signals in poultry dust and cell culture propagated virus stocks. Embryonated eggs were used as the gold standard to assess ILTV viability. The effect of inactivation using heat-treatment, freeze-thaw and disinfectant on the assessment of integrity of the ILTV structure using DNAse, PMA and IMS treatments is also presented and discussed.

## 2. Methodology

The experiments conducted are summarised in **Table 1**.

**Table 1:** Summary of experiments carried out and treatments and methods used in each experiment. Chick embryo or cell culture assays were used to titrate the infectivity of samples used. Pre-DNA extraction treatments (DNAse, PMA, IMS) were then tested to assess their ability to prevent qPCR amplification of DNA from inactivated ILTV.

Experiments	Pre-DNA extraction treatments	Sample material			Inactivation method						Test method		
		Virus stock	Field dust	Spiked dust	Heat	Freeze thaw	Heat + Freeze thaw	Virkon S	Proteinase K	Triton X	Cell culture^b^	qPCR	Egg embryo inoculation
EGG 1^a^		**✔**	**✔**	**✔**								**✔**	**✔**
EGG 2^a^		**✔**		**✔**	**✔**		**✔**					**✔**	**✔**
DNAse 1	DNAse	**✔**			**✔**	**✔**			**✔**		**✔**	**✔**	
DNAse 2	DNAse		**✔**									**✔**	
PMA 1	PMA	**✔**	**✔**		**✔**					**✔**	**✔**	**✔**	**✔**
PMA 2	PMA		**✔**	**✔**	**✔**		**✔**					**✔**	**✔**
IMS 1	IMS	**✔**	**✔**		**✔**						**✔**	**✔**	**✔**
IMS 2	IMS		**✔**			**✔**		**✔**				**✔**	**✔**
IMS 3	IMS		**✔**	**✔**	**✔**		**✔**					**✔**	**✔**

^a^Infectivity of the ILTV in field dust or spiked dust were tested in EGG- 1 and EGG-2 experiments and the same samples were used in all PMA and IMS experiments.

^b^Cell culture was used to test the inactivation of heat treated virus stocks used in DNAse 1, PMA 1 and IMS 1.

### 2.1 Virus stocks

A continuous cell line of hepatocellular carcinoma epithelial (LMH, ATCC CRL-2117) was used to propagate and titrate ILTV Serva strain (Nobilis ILT, MSD, batch n. 1529205). An immunoperoxidase monolayer assay was used to titrate the virus. Briefly, the virus stock was added to 96-well plates of LMH cells at 80% cell confluency in six replicates in a 10-fold dilution series from 10^−1^ to 10^−6^. After 72 h incubation, inoculated cells were fixed and incubated with a chicken anti-ILTV antibodies (LSBio, Seattle, USA), followed by incubation with a goat anti-chicken IgY conjugated with horse-radish peroxidase (Agrisera, Vannas, Sweden). A substrate solution of 3-amino-9-diethyl-carbazole was used to stain the plates. The median tissue culture infective dose (TCID_50_) titre was estimated by the method of Reed & Muench [18]. ILTV vaccine strain Serva passaged three times in LHM cell line (titre 2 × 10^3^ TCID_50_/ml) was used as a control sample containing viable virus in all experiments.

### 2.2 Poultry dust samples

#### 2.2.1 Dust naturally containing ILTV DNA

Dust samples collected from three poultry farms (A-1 and A-2 from Farm A; B from Farm B; and C from Farm C) as part of an ILTV vaccination monitoring study [3] were aliquoted for use in all experiments. Samples were tested positive for ILTV GC by PCR. For virus retrieval, dust was reconstituted in medium (1:50 w/v), vortexed for 2 min and clarified by a quick spin. The supernatant (200 μl, equivalent to 4 mg of dust sample) was used for the DNAse, IMS, and PMA treatments.

#### 2.2.2 Spiked samples

Dust samples known to be ILTV GC negative were spiked with cell culture propagated ILTV stock. Briefly, 70 mg of dust was spiked with 350 μl of ILTV stock containing 7.5 × 10^3^ TCID_50_. To investigate the effects of drying and freeze-thaw on virus survivability, spiked dust was subjected to three treatments: 1) immediately resuspended in 3150 μl of cell medium containing antibiotics (spiked wet dust); 2) dried at 30°C for 24 h in an oven (spiked dried dust); 3. dried as described above and subjected to two cycles of freezing and thawing (spiked freeze-thaw dust). Dried and freeze-thaw dust were resuspended in 3500 μl of cell medium. The supernatant of resuspended dust samples was used for inoculation in embryonated eggs, and for the PMA and IMS pre-treatments.

### 2.3 Processes used to inactivate ILTV

To investigate the influence of complete or incomplete inactivation methods on the virus capsid and virus epitopes as assessed by the DNAse, PMA and IMS treatments, the methods described below were tested. ILTV can be inactivated when exposed to heat for 15 min at 55^o^ C or 38^o^ C for 48 h and is sensitive to chemical disinfectants such as formalin, hypochlorite and idophor [19] and that repeated freeze-thaw may decrease the virus viability [17].

#### Heat

ILTV stocks (100–300 μl) were heated at 90°C for 2–4 h in a heat block and inactivation of virus was confirmed by passage in cell culture. After heat treatment, virus stocks were diluted 10-fold twice. Dust samples (5 mg/sample) were incubated for 2–5 days at 40^o^ C in a dry oven.

#### Virkon S

The supernatant of dust samples (200 μl, equivalent to 4 mg of dust) was incubated for 30 min with equal volume of 2% Virkon S (Antec, USA) solution freshly prepared using Milli-Q water. After incubation, the Virkon S activity was stopped using 0.5% (w/v) of sodium thiosulphate [20].

*Freeze-thaw*. Aliquots of virus stocks were subjected to 2, 4, 6 or 7 freeze-thaw cycles. Naturally ILTV DNA positive dust samples were subjected to 1 or 6 freeze-thaw cycles. For each cycle, virus stocks were stored at -20°C for at least 4 hours and thawed in an ice bath until completely thawed. Dust samples were stored at -20°C for at least 5 hours and thawed at room temperature for 3 hours.

#### Proteinase K and Triton X

ILTV stocks or dust samples were treated with proteinase K and Triton X. These treatments may lead to partial inactivation of virus or may facilitate the penetration of enzymes and dyes [21–23]. Samples were incubated with 0.2 U proteinase K per μl of sample for 30 min and inactivated with 1mM of phenyl methyl sulfonyl fluoride prior to the DNAse treatment. Triton X at a final concentration of 0.5% per sample was added with the PMA and incubated in the dark with rotation for 30 min before photoactivation.

### 2.4 Chick embryo assay for infectivity

The experiment was approved by the University of New England (UNE, Armidale, Australia) Animal Ethics Committee (AEC No. AEC18-040) and performed in accordance with animal ethics guidelines and approved protocols.

#### Egg maintenance

Fertile chicken broiler eggs were incubated at 37.5°C in humidified benchtop incubators with automatic egg turner (Professional Poultry Incubator P&I PI-24 Yueqing City Pandi Technology Co. LTD, China). Eggs were candled to check embryo viability prior to inoculation at day 10, and dead and weak embryos were discarded.

#### Sample preparation

Dust samples were reconstituted at a 1:50 w/v in cell medium containing 10% penicillin and streptomycin, vortexed for 2 min, incubated at room temperature for 1 hr, and clarified by centrifugation (4000 *g* for 10 min). The supernatant of naturally ILTV DNA positive samples were used undiluted (equivalent to 4 mg dust/egg) or diluted 1:10 prior to embryo inoculation. ILTV stocks were inoculated at a concentration of 400 ILTV TCID_50_/egg. Dust samples were spiked with the equivalent concentration of ILTV/egg.

#### Egg inoculation and harvesting

A total of 200 μl of inoculum per embryo was administered into the chorioallantoic membrane (CAM) using the top route [24]. After inoculation, eggs were not turned. Eggs were candled 48 h after virus inoculation and dead embryos were discarded. At termination on day 17, eggs were chilled overnight at 4°C prior to the allantoic fluid and chorioallantoic membrane (CAM) harvest [25]. Individual allantoic fluid samples were retrieved for ILTV PCR testing. CAM was monitored for pock lesions. Samples that produced pock lesions and contained similar or increased amounts of ILTV DNA in allantoic fluid compared to the inoculum were regarded as containing viable ILTV. Samples without pock lesions and that were ILTV DNA negative by PCR were considered as not containing viable ILTV.

#### Application of the embryonated egg method for assessing ILTV infectivity

Two experiments were carried out. The first experiment (EGG-1) evaluated if naturally ILTV DNA positive dust samples A1, A2, B and C contained viable virus. ILTV spiked dust (spiked wet dust) and ILTV stocks were used as positive controls **(Table 1).** Samples were passaged twice in embryonated eggs. For the first passage, 4–5 embryonated eggs were used per sample (n = 48). Allantoic fluids from the first passage were used as inoculum for the subsequent passage in embryonated eggs after ILTV PCR testing (n = 65). ILTV PCR negative allantoic fluids from eggs inoculated with a given sample were pooled and this pool was used to inoculate 2–3 embryonated eggs (n = 24). ILTV PCR positive allantoic fluid from individual eggs was inoculated in 2–3 embryonated eggs (n = 41).

The second experiment (EGG-2) was performed to evaluate the effect of drying and freeze-thaw cycles on ILTV survivability in dust **(Table 1).** Dust were spiked with ILTV and processed as described above to generate wet dust, dried dust and freeze-thaw dust. Virus stocks were used as positive control. Five embryonated eggs were used per sample (n = 20). The same batch of samples was used for the IMS and PMA experiments described below.

In both experiments, embryos that died within 1–3 days after inoculation were excluded from analysis.

### 2.5 DNAse treatment

Virus stocks and dust samples were treated with DNAse I (New England Biolabs, Australia) at a concentration of 0.2 or 0.5 U/μl of sample (20–50 μl of DNAse per 200 μl sample) as per manufacturer’s instructions but the incubation time was increased to 30 min. The doses of the DNAse were sufficient to digest extracts containing 68−87 ng DNA.

To evaluate the effect of DNAse treatment on qPCR amplification following inactivation two experiments (DNAse-1 and DNAse-2) with full factorial design were conducted **(Table 1).** In DNAse-1, virus stocks were subjected to 2 × heat-treatments (heat treated, non-heat treated), 4 × cycles of freeze/thaw (2, 4, 6 and 7), 2 × doses of proteinase K (0 and 0.2 U/ μl) and 2 × doses of DNAse (0 and 0.2 U/μl). In DNAse-2, 12 naturally ILTV DNA positive dust samples containing inactivated ILTV were subjected to DNAse I treatment at a concentration of 0.5 U/μl (50 μl DNAse per 200 μl of sample) or left untreated.

### 2.6 PMA treatment

Virus stocks and dust samples were treated with PMA (PMAxx^™^ Dye, 20 mM in H_2_O, Biotium, USA) at a final concentration of 100 μM per sample. Extracted DNA was used as a control. The dose was selected based on previous reports that 100 μM concentration of PMA was effective in differentiating viable and non-viable bacteriophage T4 [11] and enteric viruses [26]. To evaluate the effect of inactivation methods in the PMA-PCR, two experiments (PMA-1 and PMA-2) with full factorial design were conducted **(Table 1).**

In PMA-1, dust sample A2 and virus stock were subjected to 2 × heat-treatments (heat treated, non-heat treated), 2 × light exposure times (5 and 20 min), 2 × doses of Triton X (0 and 0.5%) and 2 × doses PMA (0 and 100 μM PMA/200 μl sample) with 2 replications. PMA treated samples were incubated in the dark for 20 min and a 500 W halogen light was used for photoactivation of the dye.

In PMA-2, ILTV DNA positive dust samples A1, B and C, and spiked dust samples (nil treatment, dried, dried and freeze-thaw) were treated with PMA (100 μM PMA/200 μl of sample) or left untreated with 2 replications. The samples were exposed to light for 5 min as described above.

### 2.7 IMS treatment

IMS was performed using Dynabeads Protein A (Thermo Fisher Scientific, USA) following manufacturer’s instruction with minor modifications. The antibody binding (ILTV glycoprotein E Polyclonal Antibody, Bioss Antibodies Inc., USA) and immunoprecipitation of the target antigen steps were increased to 30 min each. Viral antigen was eluted by heating bead suspensions at 70°C for 10 min then chilling on ice for 3 min. Eluted viral antigen was subjected to DNA extraction and the beads were removed before ethanol precipitation. To evaluate the effect of inactivation methods in the PMA-PCR, three experiments (IMS-1 to IMS-3) with full factorial design were conducted **(Table 1).**

In IMS-1, dust sample A2 and virus stock were subjected to 2 × heat treatments (heat treated, non-heat treated) and 2 × IMS treatment (with or without 5 μg and 10 μg Ab). A concentration of 5 μg of antibodies was selected for further experiments.

In IMS-2, dust sample B was subjected to 3 × cycles of freeze/thaw (0, 1 and 6), 2 × doses of Virkon S (0 and 2%) and 2 × IMS treatment (with and without).

In IMS-3, ILTV DNA naturally positive dust samples A1, B and C and spiked dust samples (nil treatment, dried, dried and freeze-thaw) were treated with IMS or left untreated with 2 replications.

### 2.8 DNA extraction and quantitative ILTV PCR

DNA was extracted from 200 μl volume of test samples using the Bioline ISOLATE II Genomic DNA kit (Cat No. BIO-52067) following manufacturer’s instructions. DNA was eluted in a final volume of 100 μl and stored at -20°C until further analysis. Extracted DNA samples were then subjected to quantitative PCR (qPCR) to determine ILTV genome copies (GC) using a real-time PCR targeting the gC gene of ILTV [27]. All amplifications were carried out using a Rotor-Gene real-time PCR instrument (Qiagen, Australia). A standard curve derived from plasmid DNA was used for absolute quantification of viral genome copy number. Results were reported in log_10_ GC per reaction.

### 2.9 Statistical analysis

Statistical analyses were performed using JMP v.14 software (SAS Institute, Cary, NC, USA). The ILTV GC were log_10_ transformed before data analysis.

To evaluate the presence of viable virus stocks in the dust samples (EGG-1), ILTV log_10_ GC of virus in allantoic fluids of eggs was analysed by fitting the inoculum used (dust samples, spiked wet dust and virus stocks), number of passage of the inoculum in eggs and their interaction as a fixed effect. Similarly, to determine the survivability of ILTV in dry dust (EGG- 2), ILTV log_10_ GC in allantoic fluid of eggs was analysed by fitting the inoculum used (spiked dry, spiked wet and spiked freeze-thaw dust, virus stocks), number of passage and their interaction as fixed effects. For all models, the effects and interactions that were not significant were removed. The overall effect of each treatment in the ILTV log_10_ GC was analysed by combining data of all experiments and fitting DNAse, PMA or IMS as a fixed effect.

To evaluate the DNAse treatment in experiment DNAse-1, ILTV log_10_ GC of virus stocks was analysed fitting DNAse (with and without), proteinase K (with and without), freeze-thaw cycles (2, 4, 6, 7), heat treatment (with and without) and their interaction as fixed effects. In experiment DNAse-2, ILTV log_10_ GC of dust was analysed fitting the effect of DNAse as fixed effect.

To evaluate the PMA treatment in experiment PMA-1, ILTV log_10_ GC of dust and virus stocks was analysed fitting PMA (with and without), Triton X (with and without), light duration (5 min and 20 min), heat treatment (with and without) and their interactions as fixed effects. In experiment PMA-2, ILTV log_10_ GC of naturally ILTV DNA positive dust samples, and spiked dust samples were analysed fitting the effect of PMA as a fixed effect.

To evaluate the IMS treatment in experiment IMS-1, ILTV log_10_ GC of dust and viral stocks were analysed by fitting heat treatment (with and without), IMS (with and without) and their interaction as fixed effects. In experiment IMS-2, ILTV log_10_ GC of dust was analysed by fitting Virkon S (with and without), freeze-thaw cycle (0, 1 and 6), IMS (with and without) and their interaction as fixed effects. In IMS-3, ILTV log_10_ GC of naturally ILTV DNA positive dust samples, and spiked dust samples were analysed fitting the effect of PMA as a fixed effect.

Data are presented as least-squares means (LSM) ± SEM. A significance level of p < 0.05 is used throughout.

## 3. Results

### 3.1 ILTV can be isolated from spiked dust but not from naturally ILTV DNA positive dust samples

**Fig 1:**
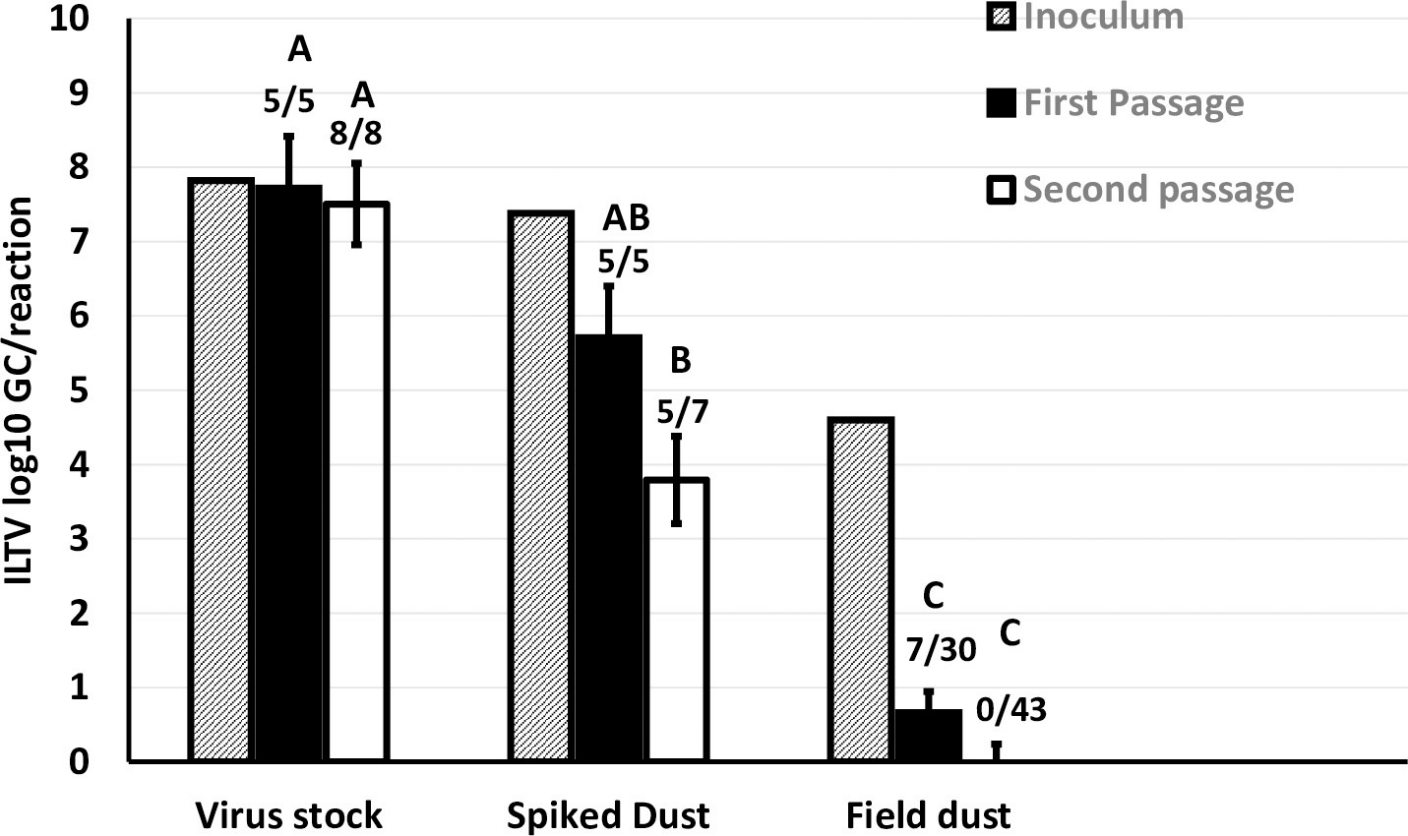
Experiment EGG-1. ILTV log_10_ GC (LSM ± SE) in the inoculum and allantoic fluid of embryonated eggs inoculated with dust samples naturally ILTV log_10_ GC positive, spiked dust and ILTV stock propagated in cell culture after the first and second passage. Number of positive samples and total number of samples are also given. Superscripts ^(ABC)^ not sharing the same letter denote difference (p <0.05) between samples.

Experiment EGG-1 investigated if naturally ILTV DNA positive dust samples contained infectious virus. ILTV propagated in cell culture and spiked wet dust samples were used as positive controls. Viable ILTV could be retrieved from eggs inoculated with virus stocks and spiked dust but not with naturally ILTV DNA positive dust (**Fig 1**). The number of ILTV GC positive samples and the concentration of ILTV GC in allantoic fluid was lower for embryos inoculated with spiked wet dust compared to the ILTV stock inoculum at the same virus concentration **(Fig 1)**, indicating a reduction in viral infectivity after contact with dust. Pock lesions were observed in 1/7 embryonated eggs inoculated with spiked dust and in 3/8 embryonated eggs inoculated with ILTV stock after the second passage. ILTV could not be isolated from the four naturally ILTV DNA positive dust samples tested as evidenced by the lack of PCR amplification in allantoic fluid and the lack of pock lesions in the CAM after two passages in embryonated eggs **(Fig 1).**

Experiment EGG-2 further investigated the survivability of ILTV in spiked dust samples subjected to drying and freeze thaw cycles compared to spiked dust that were not dried. After the first passage in embryos there was a 4 log reduction in ILTV log_10_ GC in allantoic fluid of embryos inoculated with spiked dust subjected to drying relative to those inoculated with untreated spiked samples (p < 0.05) (**Table 2**). Freezing and thawing of dried dust samples for 2 cycles did not cause further reduction in ILTV log_10_ GC. Pock lesions in the CAM were seen in 3/4 embryonated eggs inoculated with the virus stocks and 1/4 embryonated eggs inoculated with the untreated spiked dust, while no pock lesion was seen in the CAM of embryonated eggs inoculated with the dried (0/4) and dried freeze thaw (0/4) dust samples.

**Table 2:** Experiment EGG-2. ILTV log_10_ GC in the inoculum and allantoic fluid of eggs inoculated with virus stock or an equivalent amount of stock spiked on dust samples subjected or not to drying at 30°C for 24 h and two freeze-thaw cycles.

Sample	Sample treatment	LSM ± SE ILTV log_10_ GC/reaction of inoculum	LSM ± SE ILTV log_10_ GC/reaction of allantoic fluid (N. positive /total)
Virus stocks	Nil	7.62 ± 0.66^A,^^1^	(4/4) 8.81 ± 0.57^A^
Spiked dust	Nil	7.82 ± 0.66^A^	(4/4) 6.61± 0.57^A^
Spiked dust	Drying	6.88 ± 0.66^A^	(3/4) 2.49 ± 0.57^B^
Spiked dust	Drying plus freeze-thaw	6.79 ± 0.66^A^	(4/4) 3.78 ± 0.57^B^

^1^Superscripts (ABC) not sharing the same letter denote difference (p <0.05) between the samples

### 3.2 DNAse could not discriminate between viable and non-viable ILTV

**Fig 2:**
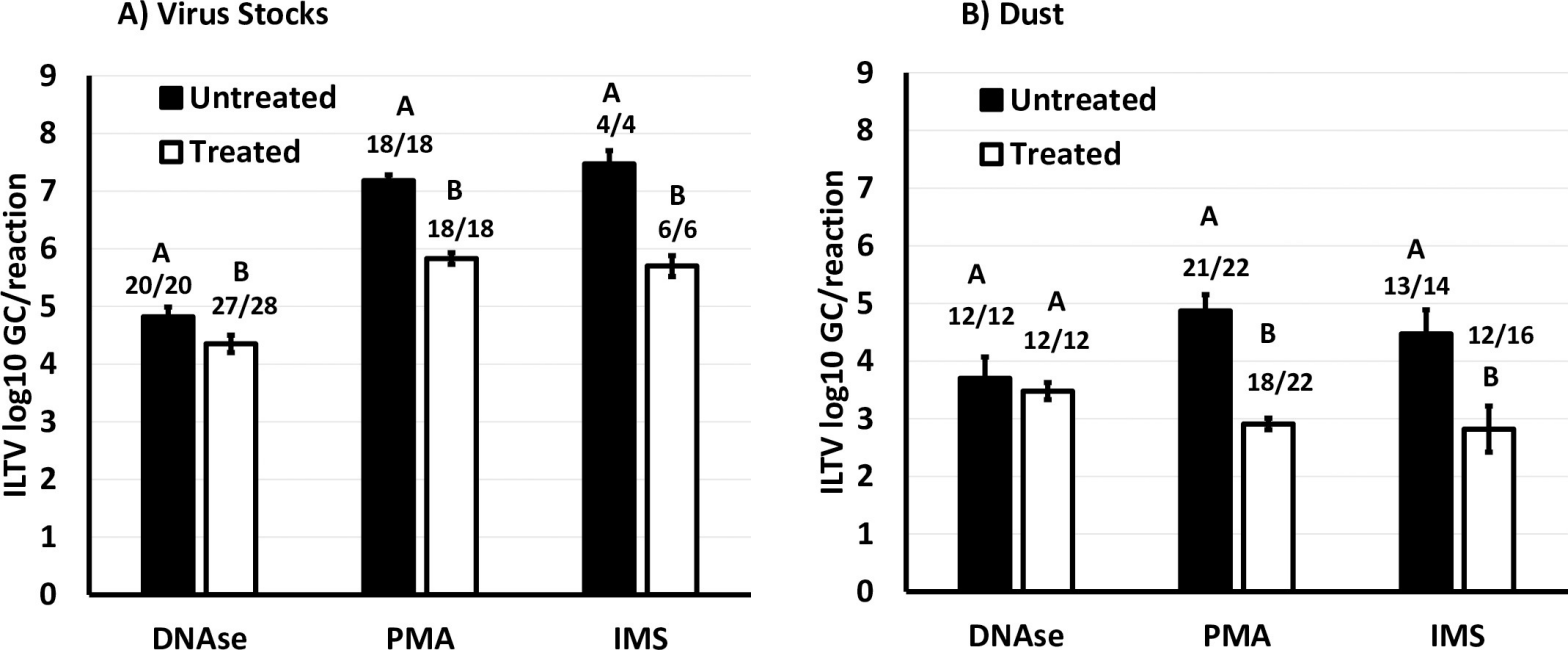
Effect of DNAse, IMS and PMA pre-treatments in ILTV GC. ILTV log_10_ GC (LSM ± SE) in DNAse, IMS and PMA treated and untreated virus stocks (A) and naturally ILTV DNA positive dust (B). Number of positive samples and total number of samples are also given. Superscripts ^(ABC)^ not sharing the same letter denote difference (p <0.05) between samples.

Overall, there was a 0.5 ILTV log_10_ GC decrease in DNAse treated virus stocks compared to untreated samples (p = 0.04) while the proportion of samples ILTV log_10_ GC positive were similar for untreated (20/20) and DNAse treated samples (27/28) (**Fig 2A**). For 12 dust samples naturally containing inactivated ILTV, DNAse treatment had no effect on the number of positive samples or ILTV log_10_ GC levels (p = 0.68) **(Fig 2B).**

When inactivation treatments were applied to virus stocks, there was no difference in the ILTV log_10_ GC of heat treated (5.20 ± 0.60) and non-heat treated (4.71 ± 0.60) (p = 0.61) stocks; or between samples treated with proteinase K (5.02±0.42) or untreated (4.96 ± 0.42) (p = 0.92). Similarly, cycles of freezing-thawing had no effect in ILTV log_10_ GC levels (p = 0.19). There was also no significant interaction between the effect of DNAse treatment and inactivation treatments (heat and proteinase K) (p = 0.28).

### 3.3 PMA treatment resulted in reduction of qPCR amplification of ILTV DNA in virus stock and dust samples but the reduction was no greater in samples containing inactivated virus

Overall, there was a 1.3 reduction in ILTV log_10_ GC in PMA treated virus stocks compared to untreated stocks (p < 0.0001) while there was no difference in the number of ILTV log_10_ GC positive samples (**Fig 2**). When virus stocks were treated with Triton X there was no effect on the ILTV log_10_ GC of Triton X treated (6.56 ± 0.10) and untreated stocks (6.39 ± 0.10) (p = 0.23). Similarly, there was no effect of heat-treatment (6.51 ± 0.10) compared to non-treatment of virus stocks (6.44 ± 0.10) (p = 0.62). Length of light exposure for photoactivation of PMA for 5 min (6.45 ± 0.10) and 10 min (6.49 ± 0.10) had no effect in ILTV log_10_ GC (p = 0.77). PMA treatment did not enable differentiation between heat treated (6.00 ± 0.14) and non-heat treated virus stocks (5.63 ± 0.14) (p ˃ 0.05). Addition of Triton X did not improve differentiation of heat treated (6.41 ± 0.20) and non-heat treated virus stocks (5.54 ± 0.20) (p ˃ 0.05).

For dust samples naturally ILTV GC positive, there was a 2 log_10_ reduction in ILTV GC levels in PMA treated samples compared to untreated ones (p < 0. 0001) with no difference in the proportion of positive samples (**Fig 2B**). When dust samples were treated with Triton X there was no effect on ILTV log_10_ GC levels (treated 4.32 ± 0.07; untreated 4.46 ± 0.07) (p = 0.19). Similarly there was no effect of length of photoactivation for 5 min (4.30 ± 0.07) or 10 min (4.47± 0.07) (p = 0.13). Contrary to the observation with virus stocks, heat treatment caused a 0.5 log_10_ reduction in ILTV GC (4.16 ± 0.7) compared to non-heat treated dust samples (4.62 ± 0.07) (p < 0.001). A similar 0.6 log_10_ reduction was seen in heat-treated dust samples subjected to PMA (3.29 ± 0.11) compared to non-heat treated dust samples (3.87 ± 0.11) (p < 0.05).

**Fig 3:**
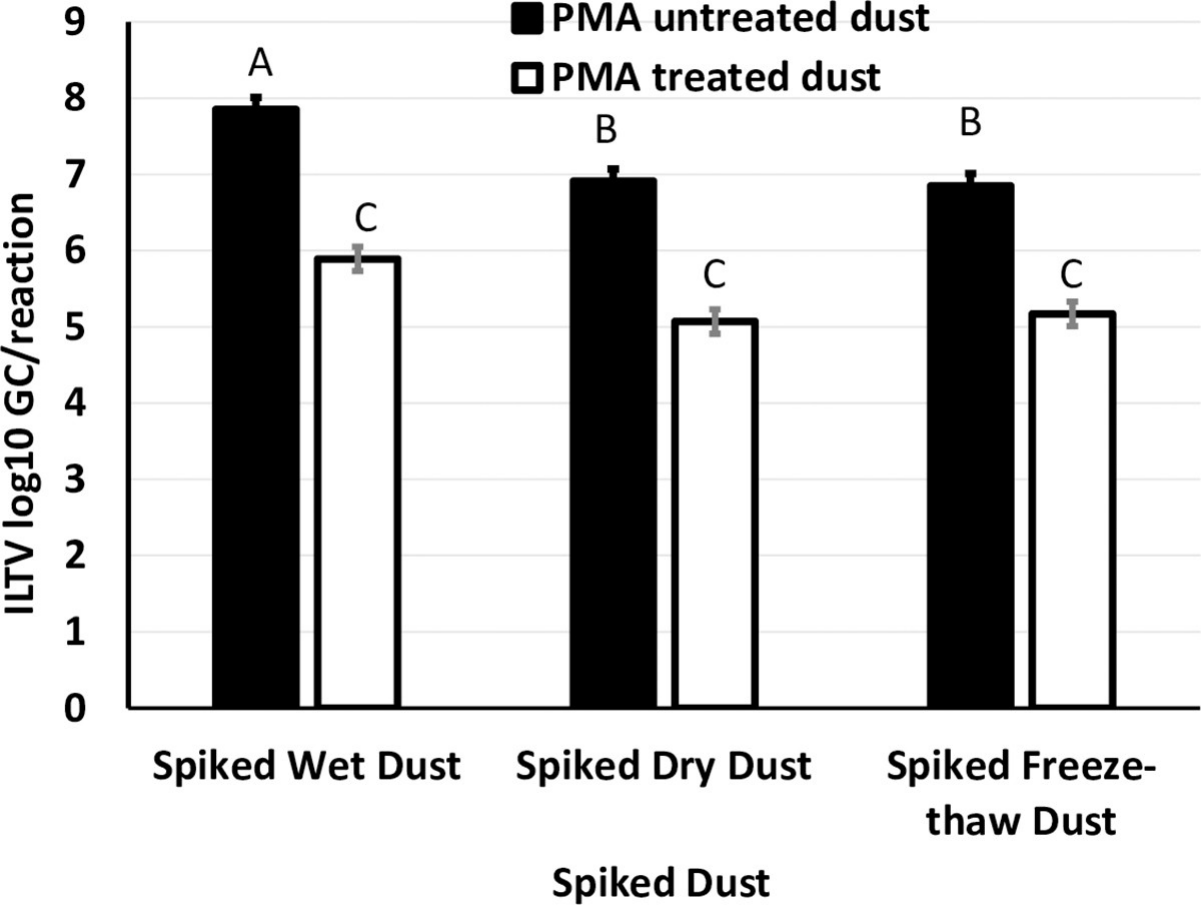
Effect of PMA pre-treatment on ILTV GC recovered from spiked dust samples subjected to three different treatments. Dust was spiked with ILTV at a concentration of 400 TCID_50_ and either reconstituted in cell medium immediately (Nil) or dried at 30°C for 24h (Dried) or dried and subjected to two freeze-thaw cycles (Dried and freeze-thaw). Superscripts (^ABC^) not sharing the same letter denote difference (p < 0.05) between samples.

For spiked dust samples, pre-treatment of samples with PMA resulted in a 2.0 log_10_ GC reduction in untreated spiked dust, 1.9 log_10_ GC in dried dust and 1.7 log_10_ GC in dried then freeze-thawed samples relative to samples not treated with PMA (**Fig 3**). All samples were qPCR positive for ILTV.

### 3.4 IMS treatment resulted in reduction of qPCR amplification of ILTV DNA in virus stock and dust samples but the reduction was no greater in samples containing inactivated virus

Overall, there was a 1.8 log_10_ GC reduction in IMS treated virus stock samples compared to untreated samples (p < 0. 001) (**Fig 2A**). When virus stocks were subjected to heat treatment there was no difference in ILTV log_10_ GC between heat treated (6.95 ± 0.24) and non-heat treated stocks (6.33 ± 0.24) (p = 0.21). Pre-treatment with IMS failed to induce differences in GC between heat treated (6.31 ± 0.28) and non-heat treated virus stocks (5.46± 0.28) (p ˃ 0.05).

For dust samples, there was a reduction of 1.7 ILTV log_10_ GC in IMS treated samples compared to untreated samples (p < 0.01) (**Fig 2B**). In dust samples subjected to inactivation treatments, there was no difference in ILTV log_10_ GC between Virkon S treated (2.78 ± 0.45) and untreated (4.18 ±0.45) dust samples (p = 0.16), and heat treated (4.98 ± 0.10) and non-heat treated (4.84 ± 0.10) dust samples (p = 0.40). Similarly, freeze-thawing cycles (0 to 6 cycles) had no effect on ILTV log_10_ GC levels (p = 0.53). Pre-treatment with IMS did not enable differentiation between Virkon S treated (1.19 ± 0.64) and untreated dust (3.44 ± 0.64) (p ˃ 0.05); heat treated (4.63 ± 0.11) and non-heat treated dust (4.22 ± 0.11) (p ˃ 0.05).

**Fig 4:**
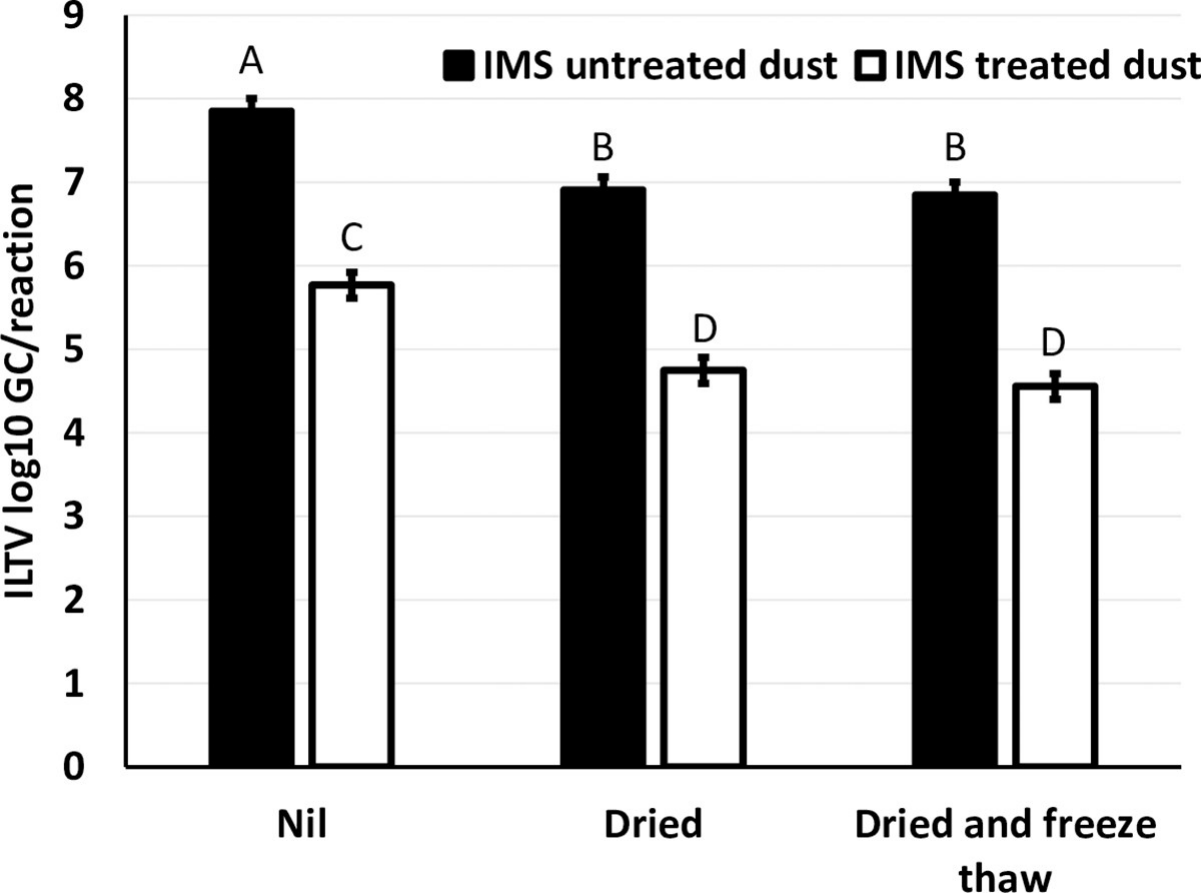
Effect of IMS pre-treatment on ILTV GC recovered from spiked dust samples subjected to three different treatments. Dust was spiked with ILTV at a concentration of 400 TCID_50_ and either reconstituted in cell medium immediately (Nil) or dried at 30°C for 24h (Dried) or dried and subjected to two freeze-thaw cycles (Dried and freeze-thaw). Superscripts (^ABC^) not sharing the same letter denote difference (p < 0.05) between samples.

For spiked dust samples pre-treated with IMS there was a reduction of 2.1 log_10_ GC in untreated spiked dust, 2.2 log_10_ GC in dried dust, and 2.3 log_10_ GC in dried then freeze-thawed relative to samples not treated with IMS **(Fig 4).** All samples were qPCR positive for ILTV.

## 4. Discussion

This study evaluated three treatments (PMA, DNAse and IMS) applied prior to nucleic acid extraction of dust samples and ILTV cell propagated stocks for their ability to prevent amplification of DNA from non-viable viral particles prior to PCR, thus enabling amplification only of viable virus. It is presumed that any reduction in amplification of ILTV DNA in samples following pre-treatment with DNAse, PMA or IMS is due to selective inhibition of amplification from non-viable virus. Of the methods tested, DNAse could not prevent the amplification of inactivated ILTV DNA from dust samples but caused a slight reduction on the amplification of ILTV from virus stocks. PMA and IMS partially prevented the amplification of ILTV DNA, but the extent of this reduction was the same for samples subjected to virus inactivation procedures or not. The lack of further reduction on the ILTV DNA amplification in samples containing only non-viable ILTV indicates that these treatments are unable to differentiate between viable and non-viable ILTV. This may be due to the inability of the inactivation treatments to sufficiently destroy the integrity of epitopes, or damage the capsid and tegument that protect viral nucleic acids.

There was a reduction in viral load in embryos inoculated with spiked dust indicating that dust has properties that inactivate the virus. Drying of the same dust sample significantly further decreased the ILTV infectivity in embryonated eggs. This indicates that desiccation can further inactivate ILTV. Any small changes in the viral protein structure can alter infectivity [28]. However, such inactivation could not be detected by PCR using the pre-extraction methods tested in this study. Further investigation is required to evaluate the infectivity of ILTV in commercial dust samples as it is assumed that outbreaks of ILTV may be caused by dispersion of dust from contaminated farms [29], which is contrary to the low viability of ILTV in dust samples observed in this study.

DNAse treatment prior to DNA extraction did not enable differentiation between heat inactivated and viable virus stocks. Similarly, DNAse had the lowest ILTV log_10_ reduction between DNAse treated and untreated dust samples. This may be due to the high molecular weight of DNAse which may have prevented effective penetration of DNAse into inactivated virus particles with damaged capsid or tegument. In the case of environmental samples, the presence of inhibitors can also hinder the DNAse activity [30]. Addition of proteinase K to the heat treatment did not improve DNAse performance in differentiating viable and nonviable ILTV stocks. A similar finding was observed by Baert et al. [31] who described only a 0.2 log_10_ reduction in infectivity of heat treated and non-heat treated murine norovirus-1 after proteinase K and RNAse treatment. However, Nuanualsuwan and Cliver [23] successfully differentiated intact hepatitis A virus, poliovirus and feline calicivirus from stocks inactivated via UV, chlorine and heat treatment at 72°C after using proteinase K and nuclease. Differences between studies may be due to differences in the viruses studied and the use of different inactivation protocols.

Although PMA performed better than DNAse in reducing PCR signals from ILTV in test materials, it could not differentiate between non-viable and viable virus stocks. This indicates that heat treatment could not sufficiently disrupt the outer structure of virus or only promoted partial disruption hindering effective penetration of PMA into inactivated virus. This is similar to what Graiver et al. [32] found when evaluating ethidium monoazide, an intercalating dye similar to PMA, on the differentiation of infective or thermally inactivated avian influenza virus. In the study conducted by Fittipaldi, Rodriguez [11], PMA could not differentiate viable bacteriophage T4 heat treated at 85°C for up to 15 min. However, there was a significant reduction of more than 7 log_10_ GC when samples were heat treated at 110°C, presumably due to sufficient capsid damage to allow PMA to penetrate into the inactivated bacteriophage. This suggests that that the outer structure protecting the nucleic acid of virus should be sufficiently disrupted for the PMA to act effectively. Similarly, the inability of the PMA treatment to completely suppress the naturally ILTV DNA positive dust samples that were known to contain non-viable ILTV shows that natural inactivation of ILTV may occur without significant damage of the outer structures. Interestingly, the heat treatments applied to naturally ILTV GC dust positive samples in our study also had no to modest effects on the reduction of ILTV GC load, indicating the inactivation treatments used were not able to sufficiently damage the outer structures of the virus in dust, as was observed with virus stocks in liquid medium.

IMS, which assesses the virus inactivation based on the loss of integrity of epitopes, could not differentiate between heat, or Virkon S treated and untreated ILTV in dust or virus stocks. A polyclonal antibody targeting the envelope glycoprotein E was used as this protein is essential for cell to cell spread of ILTV [33]. Generally enveloped viruses are considered to be more susceptible to chemical treatments due to the presence of the easily degradable lipid envelope, however, information on the mode of action of specific chemical disinfectants in denaturizing the antigenic epitopes or envelope of enveloped viruses are not available [34]. It is possible that the Virkon S or heat treatment used in this experiment did not completely degrade the glycoprotein E or that the organic materials present in dust altered the action of the disinfectant by neutralization or hindering its direct contact with the virus [34]. Alternatively, the polyclonal antibody used cross-reacted with the other antigenic epitopes present in capsid protein or tegument yielding false positive results. Further investigation regarding the stability of epitopes, glycoprotein and envelope of ILTV in response to different inactivation agents using electron microscopy and use of use of specific monoclonal antibody to conduct IMS would provide better understanding of nature of virus inactivation.

Under the conditions of this study, none of the methods tested provided practical means to differentiate inactivated or infectious ILTV in dust samples, which was the main objective of this study. Further optimisation of PMA and IMS may help to improve the efficiency of these techniques such as increasing the dose and incubation time of PMA, repeatedly exposing samples to PMA [11, 35] or combining PMA and IMS treatments.

## 5. Conclusions

In conclusion, the use of pre-DNA extraction treatments to block qPCR amplification of DNA from non-viable ILTV in poultry dust and virus stock using integrity of virus outer structure (DNAse, PMA) or antigenic structure (IMS) as markers of virus infectivity failed to block amplification from non-viable virus and thus overestimated the amount of viable ILTV. DNAse treatment did not reduce the PCR signals of inactivated ILTV genome in dust samples but a small reduction was observed in virus stocks. PMA and IMS reduced PCR signals of ILTV genome from dust and virus stocks by approximately 100-fold on average, however, this reduction was non-discriminatory of inactivated and non-inactivated samples.

## Supporting information

S1 Data (XLSX)
